# Clinical Pilot Series of Non-Self-Contained Periodontal Infrabony Defects Treated with a Slowly Resorbable Bovine Pericardium Membrane in Combination with Low-Temperature-Treated Decellularized Bovine Bone Particles

**DOI:** 10.3390/dj9100110

**Published:** 2021-09-26

**Authors:** Mariana A. Rojas, Lorenzo Marini, Paola Russo, Vittorio Blardi, Patrick R. Schmidlin, Andrea Pilloni

**Affiliations:** 1 Section of Periodontics, Department of Oral and Maxillofacial Sciences, Sapienza University of Rome, 00161 Rome, Italy; marianaandrea.rojas@uniroma1.it (M.A.R.); paolarusso8484@tiscali.it (P.R.); vittorio93_blardi@hotmail.it (V.B.); andrea.pilloni@uniroma1.it (A.P.); 2 Clinic of Conservative and Preventive Dentistry, Center of Dental Medicine, Division of Periodontology & Peri-Implant Diseases, University of Zurich, 8032 Zürich, Switzerland; patrick.schmidlin@zzm.uzh.ch

**Keywords:** guided tissue regeneration, osseous defects, periodontal regeneration, bone graft(s)

## Abstract

The aim of this case series was to present the clinical outcomes of non-contained intrabony periodontal defects (IPDs) treated by means of papillary preservation flaps in association with a slowly resorbable bovine pericardium membrane (BPM) and a low-temperature-treated bovine bone graft (BBG). Eight healthy, non-smoking patients (two males and six females, mean age 48 ± 8 years) with stage 3 periodontitis and at least one site with residual probing depth (PD) ≥ 6 mm associated with a non-contained IPD ≥ 3 mm were treated. Two weeks after surgery, no adverse events were observed, and an early wound healing score (EHS) of 8.1 ± 1.0 was recorded. After 1 year, the mean probing depth (PD) reduction and mean clinical attachment level gain (CAL-gain) accounted for 4.8 ± 0.7 and 3.5 ± 0.7 mm, respectively, whereas the mean gingival recession (REC) was of 1.2 ± 0.3 mm. Radiographic bone fill was observed in all cases. In conclusion, the treatment of non-contained IPDs with a slowly resorbable BPM and a low-temperature-treated BBG could be considered safe and may result in significant clinical improvements 1 year after surgery.

## 1. Introduction

The goal of periodontal therapy is to avoid further progression of the periodontal breakdown, preventing the loss of teeth and masticatory function. This is achieved through the reduction or elimination of gingival inflammation and of deep periodontal pockets and the regain of periodontal attachment [1,2].

Infrabony periodontal defects (IPDs) are an anatomical form of periodontal destruction and, associated with periodontal pockets, represent a higher risk of attachment loss if left untreated [3]. After oral hygiene instructions, biofilm and calculus removal (initial or cause-related therapy) with or without adjunctive anti-microbials, IPDs often require surgical intervention. In this context, better results have been demonstrated with periodontal regenerative therapies when compared to open flap debridement alone [4], and a recent systematic review has concluded that enamel matrix derivatives (EMD) or guided tissue regeneration (GTR) in combination with papillary preservation flaps (PPFs) should be considered the treatment of choice for residual pockets with deep IPD ≥ 3 mm [5].

The anatomy of the periodontal defect and the design of the flap chosen to expose the defect are essential in the selection of regenerative materials and in the surgical strategy [6]. In the presence of a non-containing defect for which a large flap has been performed, the stability of the area can be achieved by using grafting materials and membranes. According to Stavropoulos et al. [7], the use of bone fillers in association with membranes results in better outcomes on a long-term follow-up.

Based on their origin, bone fillers can be divided into autograft, allograft, xenograft and alloplast. Xenografts are biomaterials of animal origin, typically bovine, equine or porcine. Although many types of bone substitutes are commercially available, the main disadvantage in the use of these biomaterials is represented by the unpredictable level of regeneration and reabsorption. One of the main distinguishing characteristics of the xenograft, which affects the properties of the biomaterial, is the method and the temperature of processing. While most bovine xenografts have very high heating temperatures (from 300 °C to 1250 °C) which do not allow to preserve the porosity of the material that undergoes ceramization, a bovine bone filler has recently been introduced with a treatment at low temperature (−80 °C to 121 °C) [8]. Bovine bone graft (BBG) treated at low temperature could solve problems that are derived from processing at high temperature, obtaining decellularized bone particles with a higher biocompatibility, volume maintenance, better stability and absence of ceramization, with total resorption of the raw material and the preservation of the main bone markers [9].

Membranes are basically used to select the cell types involved in the regeneration process and to improve wound stability [10]. Traditionally, they are distinguished into non-resorbable and resorbable and according to their origin (autogenous, xenogeneic, allogenic and alloplastic). The resorbable membranes can be synthetic (more predictable resorption pattern) or xenogenic (more biocompatible). The natural barriers are constituted by collagen (mostly type I) and could be cross-linked to increase the resorption time. Compared to the non-resorbable, they offer several advantages, such as decrease in the numbers of surgical procedures and a greater ease of handling [11]. However, there are also some disadvantages, including an unpredictable degree of resorption depending on the degradation process (hydrolytic or enzymatic) and the possibility of an inflammatory process due to the resorption process [12]. The majority of the commercially available resorbable barriers are degraded in 4–8 weeks [8]. In the last years, a new bovine pericardium membrane (BPM) has been presented with a slow resorption rate. In fact, in addition to being produced with an innovative decellularization method that allows to maintain its collagen structure, it has been subjected to a cross-linking process that allows this barrier to stand out from the other membranes (whose collagen generally derives from other tissues or other animal species) to have a reabsorption time of 3–6 months. This gives the barrier the superior performance of non-resorbable membranes, with the advantage of being totally resorbable.

The use of the above-mentioned recently introduced BBG and BPM has been documented in the socket preservation and maxillary sinus augmentation procedures, showing excellent clinical and histomorphometric outcomes after six months [9,13].

This pilot case series presents the first clinical results after a one-year follow-up of a combination of a slowly resorbing bovine pericardium membrane and decellularized bovine bone particles treated at low temperature in the surgical treatment of non-self-contained IPDs. As primary outcome and success parameter, we define defect resolution and adequate healing as follows: no probing depth (PD) > 4 mm, absence of bleeding and a clinical attachment gain (CAL-gain) of ≥ 2 mm.

## 2. Case Series

### 2.1. Patient Information

Eight consecutive non-smoking, healthy patients (two males and six females, mean age 48 ± 8 years) affected by stage 3 periodontitis were treated at the Section of Periodontics of the Department of Oral and Maxillofacial Sciences of Sapienza University of Rome. After 6 weeks from the completion of step 1 and step 2 of periodontal therapy, as described by the clinical practice guideline of the European Federation of Periodontology [14], subjects who exhibited at least one site showing residual probing depth (PD) ≥ 6 mm associated to a two-wall non-self-containing intrabony defect ≥ 3 mm, full-mouth plaque score (FMPS) and full-mouth bleeding score (FMBS) ≤ 20% were subjected to periodontal regenerative surgery by means of papilla preservation technique in conjunction with BPM and BBG.

Before surgery, the benefits, risks, consequences of non-treatment and alternative treatment options were discussed and written informed consent was obtained from all subjects involved in the study. The study was conducted in accordance with the Declaration of Helsinki, and the protocol was approved by the Department of Oral and Maxillofacial Sciences, Sapienza, University of Rome (protocol identifying number: 699; date of approval: 24 May 2018).

### 2.2. Clinical Findings

**Figure 1:**
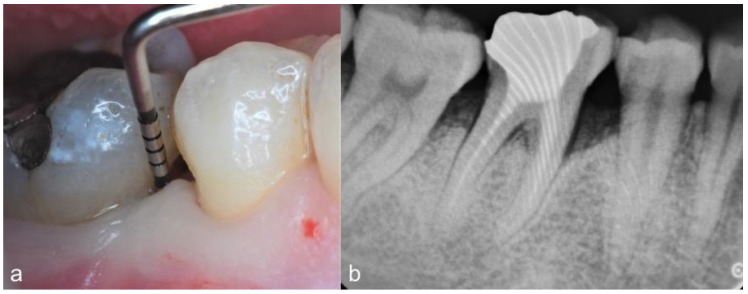
Case 7. Infrabony periodontal defect located at the mesio-buccal aspect of the first mandibular right molar. (**a**) Baseline clinical view showing an initial PD of 9 mm; (**b**) preoperative radiographic view.

Clinical parameters were recorded at baseline and 12 months postoperatively by one calibrated examiner (VB) using a calibrated periodontal probe (PCP-UNC 15, Hu-Friedy, Chicago, IL, USA) (Figure 1a,b). They included bleeding on probing (BOP), PD, gingival recession (REC) and clinical attachment level (CAL, [PD + REC]) measurements. Early wound healing score (EHS) was used to assess the clinical wound healing 14 days after surgery [15]. The EHS was based on the evaluation of clinical signs of re-epithelialization (0, 3 or 6 points), hemostasis (0, 1 or 2 points) and inflammation (0, 1 or 2 points). The sum of these three parameters calculates the EHS, which ranges between 0 (worst possible wound healing) to 10 points (ideal wound healing).

### 2.3. Therapeutic Intervention

**Figure 2:**
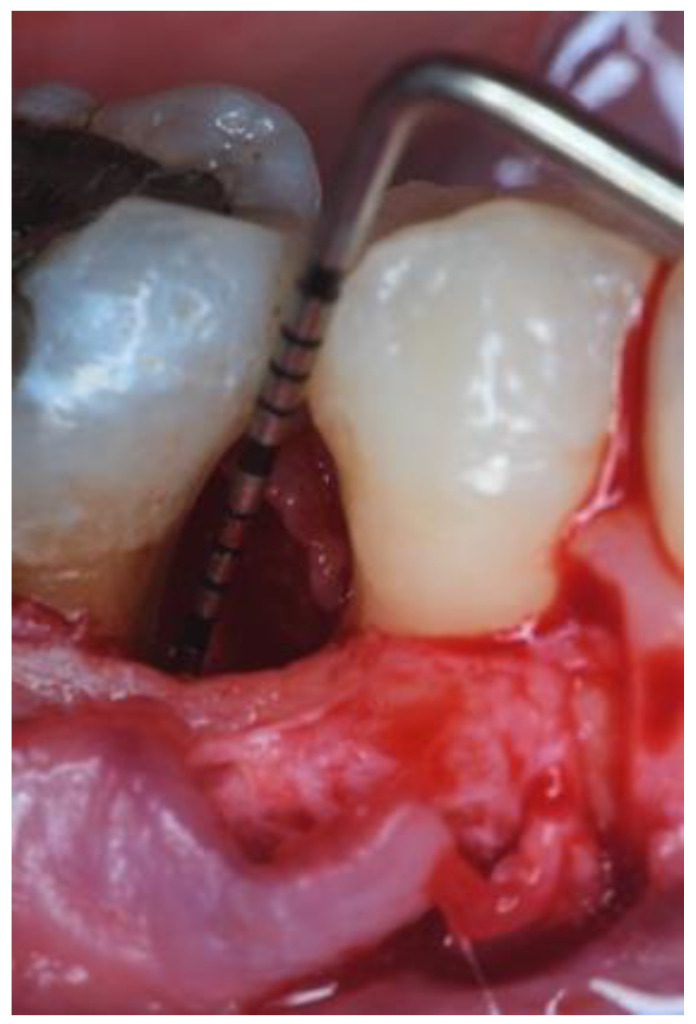
Flap elevation. Intraoperative view of the defect showing an infrabony component of 3 mm.

One experienced operator (AP) performed all the interventions. All teeth and their associated IPDs were treated with the same surgical approach. After local anesthesia, buccal and lingual intrasulcular incisions with an extension of at least one tooth mesial and distal to the defect site were performed and mucoperiosteal flaps were elevated. Modified or simplified papilla preservation technique (MPPT/SPPT) was used on the basis of the interdental space width [16,17]. Vertical releasing incisions extending into the alveolar mucosa were performed as needed to ensure proper access to the defect. After flap reflection, defect debridement and scaling and root planning were performed with hand and ultrasonic instruments. The surgical area was rinsed with sterile saline (Figure 2).

**Figure 3:**
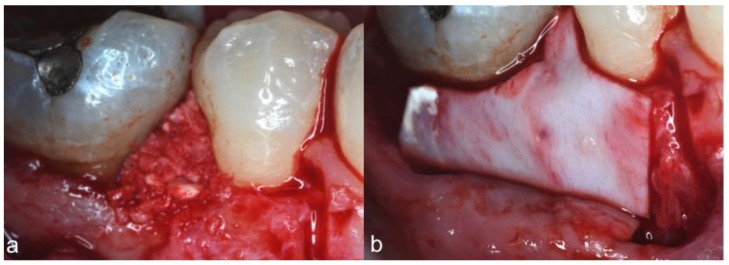
(**a**) The defect was filled with decellularized bovine bone particles; (**b**) the defect was covered completely with a bovine pericardium membrane that was trimmed, adapted and placed so as to completely cover the grafted area and the adjacent 2–3 mm of bone tissue.

BBG (RE-BONE^®^, Ubgen, Padua, Italy) was placed into the defect (Figure 3a) and BPM (SHELTER^®^ Slow membrane, Ubgen, Padua, Italy) was applied to completely cover the grafted area and the adjacent 2–3 mm of bone tissue (Figure 3b).

**Figure 4:**
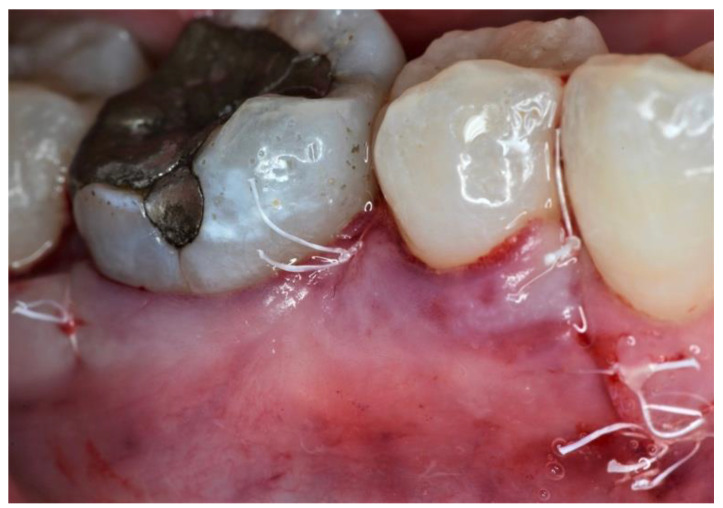
Mucoperiosteal flaps were repositioned coronally, completely covering the membrane, and were stabilized with sutures.

After periosteal release, flaps were coronally advanced, covering completely the barrier membrane and securing adequate interproximal closure (Figure 4). Suture technique (6-0 VICRYL, Ethicon, Johnson & Johnson, Somerville, NJ, USA) was performed on the basis of the flap design, according to the indications proposed by the authors, i.e., MPPT: a horizontal internal mattress suture between the base of the palatal papilla and the buccal flap and a vertical internal mattress suture between the buccal aspect of the interproximal papilla and the most coronal portion of the buccal flap [16]; SPPT: a horizontal internal mattress suture in the defect-associated interdental space and interrupted (narrow interproximal space–thin interdental tissues) or internal vertical oblique mattress suture (wide interproximal space–thick interdental tissues) on the interdental space above the membrane [17].

All patients received amoxicillin twice daily for 6 days (Zimox, Pfizer Inc., New York, NY, USA) and ibuprofen twice daily for 3 days (Brufen 600 mg, Mylan, Milano, Italy and were instructed to discontinue tooth brushing; instead, patients were asked to rinse two times/day with a 0.12% chlorhexidine gluconate solution (Periodex, Zila Pharmaceuticals Inc., Phoenix, AZ, USA) for four weeks. Sutures were removed after 14 days. After 30 days from surgery, patients were instructed to use a soft toothbrush (Gum Delicate Post-Surgical Toothbrush, Sunstar Americas Inc., Schaumburg, IL, USA). Conventional oral hygiene was resumed after three months.

### 2.4. Follow-Up and Outcomes

**Figure 5:**
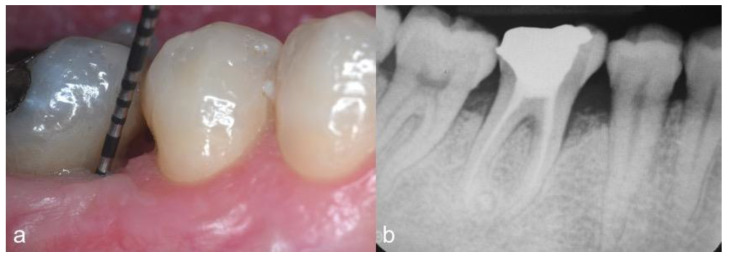
(**a**) 12-month follow-up clinical view showing significant PD reduction (residual PD of 3 mm); (**b**) 12-month follow-up periapical radiographic view showing filling of the infrabony component of the defect.

Four single-rooted (anterior) and four multi-rooted (posterior) teeth were included in this prospective pilot case series. No adverse events and post-surgical complications were observed. Two weeks after surgery, the mean value of EHS accounted for 8.1 ± 1 (range from 7 to 10). After one year, residual pockets with PD of 5 mm and 4 mm were recorded in two and four patients, respectively. Only one site (with PD = 5 mm) showed bleeding on probing. Concerning the gingival recession (REC), the mean value was of 1.2 ± 0.3 mm. Except in one case, an attachment level gain of at least 3 mm was obtained, while radiographic bone filling was observed in all cases (Figure 5b). In summary, the treatment of IPDs by means of papilla preservation flaps in association with slowly resorbable BPM + low-temperature-treated BBG resulted in a mean PD reduction of 4.8 ± 0.7 mm and CAL-gain of 3.5 ± 0.7 mm. All clinical results of all cases are presented in Table 1.

## 3. Discussion

The present clinical pilot case series on the surgical treatment of two-wall non-self-contained IPDs by means of papillary preservation flaps in association with slowly resorbable BPM and low-temperature-treated BBG showed an overall clinical success in both the anterior and posterior teeth in terms of PD reduction and CAL gain. These results are comparable with those obtained in previous studies evaluating GTR alone or the combination of other types of bone graft with barrier membranes [5,7].

Several studies evaluated different resorbable membranes in the treatment of periodontal defects [18,19]. Effectiveness of resorbable membranes has been compared with that of the non-resorbable membranes (e-PTFE), considered the ‘‘gold standard’’ [20,21,22]. The aforementioned studies concluded that resorbable membranes may be regarded as a useful alternative to e-PTFE membranes in the IPDs surgical treatment. In fact, although clinically significant CAL-gain can be obtained with GTR procedures using both bioresorbable and non-resorbable membranes, patient morbidity is lower when bioresorbable membranes are used [18]. Accordingly, in recent years, an increased interest in the use of resorbable membranes has been observed.

Since it has been shown that the wound clot must be kept stable in the first healing period in order to promote regeneration [23], the membrane resorption rate should be slow, varying between 3 and 12 months, depending on the defect anatomy [24,25,26]. From this point of view, the behavior of the membrane used in this study seems to present the ideal characteristics, allowing excellent clinical results.

In these cases, the defect anatomy and the flap design also required the use a bone substitute. For this reason, a low-temperature-treated bovine bone graft was used. The development of this processing method has been motivated by the need to improve the biocompatibility, preserving the bone’s native macro and micro porosity [27]. The BGG granules treated in this way showed microfractures that allow the cells and blood vessels to colonize the graft deeply to shorten the resorption time. Clinical and histological effectiveness of slowly resorbable BMP and low-temperature-treated BBG has been demonstrated in maxillary sinus augmentation and socket preservation procedures [9,13].

This is the first case series in the literature using the above-mentioned biomaterials in the IPD treatment, showing notable clinical and radiographic improvements at 1 year. Although a recent systematic review [28] reported flap dehiscence in 30% of the treated sites with GTR procedures using resorbable membranes, no post-surgical complications were observed. This event was also highlighted by the high mean values of EHS [15] (8.12 ± 0.99) recorded at 14 days after surgery. The radiographic bone fill was observed in all treated cases. However, radiographic analysis was not performed since the radiographs were not taken in a standardized way; therefore, an accurate analysis was not possible. Furthermore, it has been demonstrated that conventional radiographs present limitations regarding bone gain assessment [29,30]. In fact, in several studies evaluating the regenerative surgical treatment of IPDs, the radiographic analysis was not performed [29,30,31,32,33]. In this study, the intra-oral radiographs are only used to illustrate the clinical outcomes, highlighting the clinical relevance of the treatment provided.

Despite the promising findings of this pilot investigation, further randomized controlled clinical and histological trials should be performed to confirm the potential advantages of this approach compared with those of similar biomaterials.

## Figures and Tables

**Table 1:** Clinical parameters after one year, separated by tooth position.

Case	Tooth	Site	Baseline				Follow-Up				
			PD (mm)	REC (mm)	CAL (mm)	BoP (+/−)	PD (mm)	REC (mm)	CAL (mm)	BoP (+/−)	EHS
Anterior teeth											
1	1.3	DV	8	3	11	−	4	3	7	−	8
2	1.1	MP	10	0	10	+	4	2	6	−	9
3	1.1	DP	8	0	8	−	4	1	5	−	8
4	1.1	MV	6	0	6	−	3	1	4	−	10
Posterior teeth											
5	4.6	DV	9	0	9	+	5	1	6	+	7
6	4.6	ML	11	0	11	+	4	2	6	−	8
7	4.6	DV	9	0	9	−	5	1	6	−	7
8	4.6	MV	9	0	9	+	3	2	5	−	8

D, distal; M, mesial; V, vestibular; P, palatal; PD, probing depth; CAL, clinical attachment level; REC, recession depth; BoP, bleeding on probing; EHS, early wound healing score.

## Data Availability

The data presented in this study are available on request from the corresponding author.
